# Proton pump inhibitor as an independent factor of progression of abdominal aortic calcification in patients on maintenance hemodialysis

**DOI:** 10.1371/journal.pone.0199160

**Published:** 2018-07-03

**Authors:** Teppei Okamoto, Shingo Hatakeyama, Shogo Hosogoe, Yoshimi Tanaka, Kengo Imanishi, Toru Takashima, Fumitada Saitoh, Tadashi Suzuki, Chikara Ohyama

**Affiliations:** 1 Department of Urology, Oyokyo Kidney Research Institute Aomori Hospital, Aomori, Japan; 2 Department of Urology, Hirosaki University Graduate School of Medicine, Aomori, Japan; 3 Department of Urology, Oyokyo Kidney Research Institute, Aomori, Japan; Nagoya University, JAPAN

## Abstract

**Backgrounds:**

Proton pump inhibitors (PPIs) can be associated with vascular calcification in patients undergoing dialysis through hypomagnesemia. However, only few studies have demonstrated the influence of PPIs on vascular calcification in patients on maintenance hemodialysis (HD). This study aimed to investigate whether the use of PPIs accelerates vascular calcification in patients on HD.

**Materials and methods:**

We retrospectively evaluated 200 HD patients who underwent regular blood tests and computed tomography (CT) between 2016 and 2017. The abdominal aortic calcification index (ACI) was measured using abdominal CT. The difference in the ACI values between 2016 and 2017 was evaluated as ΔACI. Patients were divided into PPI and non-PPI groups, and variables, such as patient background, medication, laboratory data, and ΔACI were compared. Factors independently associated with higher ΔACI progression (≥ third tertile value of ΔACI in this study) were determined using multivariate logistic regression analysis.

**Results:**

The PPI and non-PPI groups had 112 (56%) and 88 (44%) patients, respectively. Median and third tertile value of ΔACIs were 4.2% and 5.8%, respectively. Serum magnesium was significantly lower in the PPI (2.1 mg/dL) than in the non-PPI (2.3 mg/dL) group (*P* <0.001). Median ΔACI was significantly higher in the PPI (5.0%) than in the non-PPI (3.8%) group (*P* = 0.009). A total of 77 (39%) patients had a higher ΔACI. Multivariate analysis revealed that PPIs (odds ratio = 2.23; 95% confidence interval = 1.11–4.49), annual mean calcium phosphorus product, ACI in 2016, baseline serum magnesium levels, and HD vintage were independent factors associated with higher ΔACI progression after adjusting for confounders.

**Conclusion:**

PPI use may accelerate vascular calcification in patients on HD. Further studies are necessary to elucidate their influence on vascular calcification.

## Introduction

Proton pump inhibitors (PPIs) have widely been used by the general population and patients on maintenance hemodialysis (HD) as an effective treatment against peptic ulcers and gastroesophageal reflux disease [1–3]. PPIs undergo hepatic metabolism; therefore, dosage adjustment is not necessary in patients with chronic kidney disease (CKD). However, several studies have reported an association between long-term PPI use and hypomagnesemia [3, 4]. Moreover, hypomagnesemia is associated with more severe vascular calcification [5] and higher mortality not only in healthy individuals but also patients on maintenance HD [6, 7]. Food and Drug Administration of the United States of America published a Drug Safety Communication on March 2, 2011 [8], stressing the risk for hypomagnesemia due to long-term PPI use.

A cross-sectional study reported the association between long-term PPI treatment and vascular calcification in patients on maintenance HD [9]. Moreover, another recent study suggested that PPIs potentially affect vascular endothelial cells [10]. However, whether PPI use is related with the progression of vascular calcification remains unclear. The present study therefore investigates the association between PPI use and progression of aortic calcification in patients on maintenance HD.

## Materials and methods

### Ethics statement

This was a retrospective, single center, observational study conducted in accordance with the ethical standards of the Declaration of Helsinki and approved by the Ethics Committee of Hirosaki University Graduate School of Medicine (authorization number 2017–089). The participants provided verbal informed consent, which was recorded in their medical charts. According to the provisions of the ethics committee and the ethic guideline in Japan, written consent was not needed in the case of retrospective and/or observational study using a material such as the medical records. The ethics committees of Hirosaki University School of Medicine permitted this consent procedure. The investigation information was open for the public disclosure at http://www.med.hirosaki-u.ac.jp/~uro/html/IRB/IRBdoc.html. All procedures performed in studies involving human participants were in accordance with the ethical standards of the institutional and/or national research committee and with the 1964 Helsinki declaration and its later amendments or comparable ethical standards. The trial is registered in the UMIN Clinical Trials Registry UMIN000029817

### Patient selection

**Fig 1:**
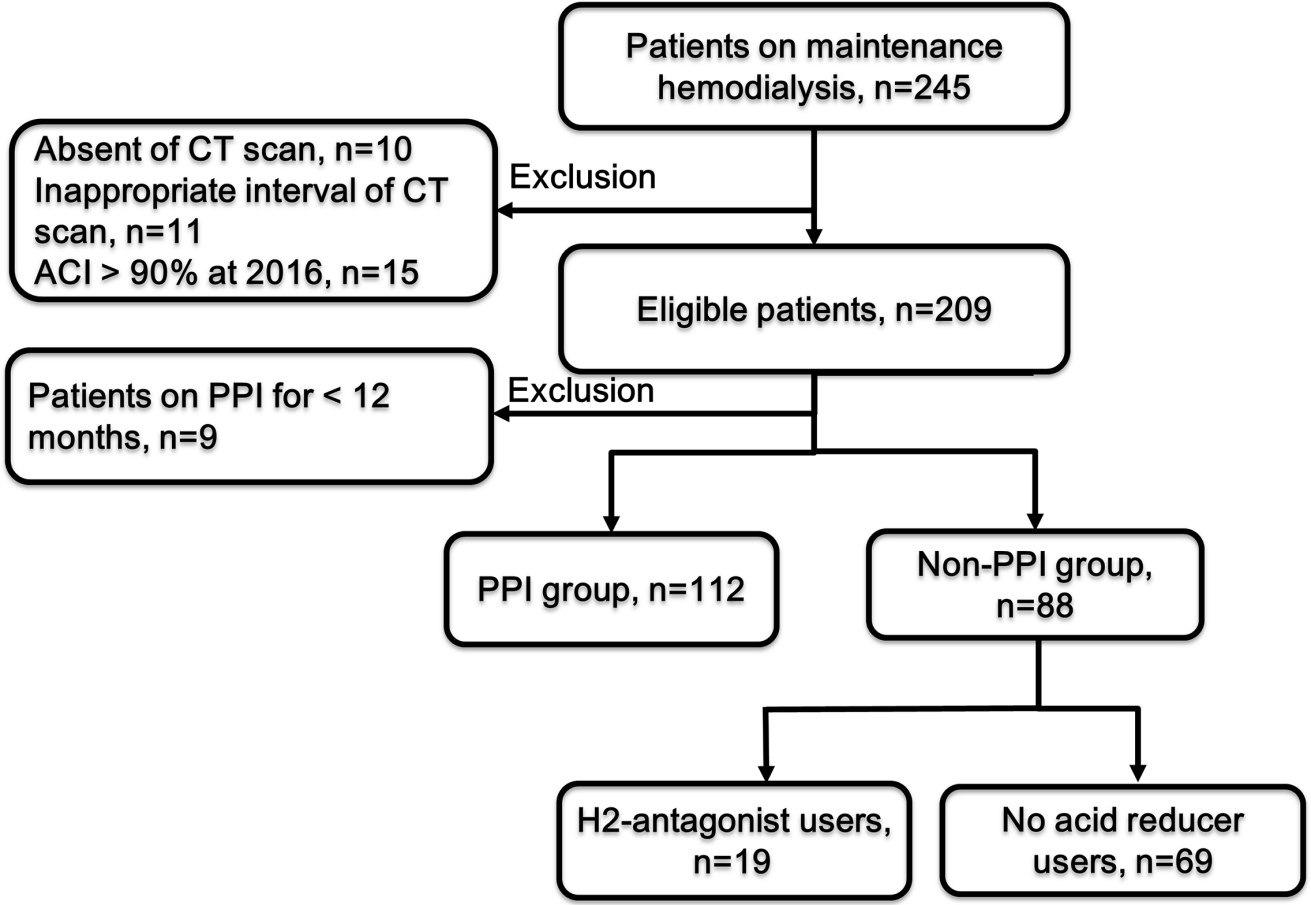
Patient selection and classification. We treated 245 hemodialysis (HD) patients who underwent 3–4 h of HD sessions thrice a week. Among these patients, 45 patients were excluded from the analyses. Ultimately, 200 patients on HD were included and divided into the proton pump inhibitor (PPI) and non-PPI groups.

Between April 2016 and September 2017, 245 patients underwent maintenance HD or hemodiafiltration for 3–4 h using dialysate containing 3.0 mEq/L calcium (Ca) and 1.0 mEq/L magnesium (Mg) thrice a week at the Oyokyo Kidney Research Institute, Aomori, Japan. Almost all patients underwent annual abdominal computed tomography (CT) for determining the presence of incidental renal tumors and other cancers. Among these patients, 16 with severe aortic calcification, including calcification extending along the entire length and almost entire circumference of the abdominal aorta, were excluded due to difficulty in evaluating the changes in aortic calcification. Moreover, 10 patients who did not undergo abdominal CT and 11 who had inappropriate intervals between subsequent CT scans (< 10 or > 15 months) were excluded. Patients who received PPIs for < 12 months during the study period were also excluded from this study. Finally, 200 patients were investigated (Fig 1).

### Evaluation of variables

Medication information [PPIs, histamine-2 receptor (H2) antagonist, antiplatelet agents, phosphate binders, warfarin, diuretic agents, and cinacalcet] was obtained from prescription records. PPIs included omeprazole, lansoprazole, rabeprazole, and esomeprazole, whereas famotidine was used as the H2-antagonist. PPI users were defined as patients who received PPIs for at least ≥12 months during the study period, whereas non-PPI users were defined as patients who received H2-antagonists or no acid reducers. Antiplatelet agents included aspirin, clopidogrel, ticlopidine, cilostazol, eicosapentaenoic acid, beraprost, and sarpogrelate. Phosphate binders included calcium carbonate, lanthanum carbonate, polymeric phosphate binders (sevelamer or bixalomer), and iron-based phosphate binders (ferric citrate hydrate or sucroferric oxyhydroxide). Furosemide was used as a diuretic agent. Patients were grouped into those with diabetic nephropathy (DMN) and others according to the cause of CKD. Current smoking was defined as smoking at least one cigarette during the observational period. A history of cardiovascular disease (CVD) was defined as any previous description of a cerebrovascular accident, ischemic heart disease, or peripheral arterial disease recorded in the patients’ medical records. This information was collected by a single examiner who was blinded from patients’ aortic calcification status.

Every month, each patient underwent a routine laboratory examination before receiving HD treatment. Laboratory data for serum levels of phosphate, albumin, Ca, Mg, C-reactive protein (CRP), and intact parathyroid hormone (i-PTH) between April 2016 and September 2017 were obtained. Baseline values of laboratory data were defined as laboratory data obtained in the month abdominal CT was conducted. We calculated annual mean values of laboratory data obtained during the observation period. The mean blood pressure from 12 dialysis sessions had been used as the representative blood pressure value during blood and serum testing. The normalized dialysis dose (Kt/V) was calculated using the following formula: Kt/V = −Ln (Ct/Co − 0.008 × t) + (4 − 3.5 × Ct/Co) × ΔBW/BW. Ct represents post-dialysis serum nitrogen; Co, pre-dialysis serum nitrogen; t, dialysis time; and W (kg), post-dialysis body weight. Serum Ca level was corrected using the following formula if the serum albumin level was < 4.0 g/dL: corrected Ca = total Ca + 0.8 × (4 − serum albumin). Calcium phosphorus product (Ca × P) was calculated by multiplying corrected calcium with serum phosphate level.

### Abdominal aortic calcification index (ACI)

Abdominal aortic calcification was measured using CT (SOMATOM Perspective, Siemens Healthineers, Tokyo, Japan). Images were obtained using a 5-mm slice thickness. Abdominal aortic calcification was semiquantitatively evaluated using CT images of the area above the common iliac artery bifurcation by performing 10 scans at 5-mm intervals as previously described [11]. Measurements of abdominal aortic calcification in 2016 and 2017 were performed simultaneously. ACI (%), which represents the calcification proportion in 12 sectors, was calculated using the following formula: ACI (%) = (total score for calcification on all slices)/12/10 × 100. Severe aortic calcification was defined as an ACI value of > 90%. The difference in ACI values between 2016 and 2017 (ΔACI) was calculated by subtracting the ACI value in 2017 (ACI-2017) from that in 2016 (ACI-2016). All procedures were conducted by a single examiner who was blinded from patients’ clinical data and background information during the measurement period. To confirm measurement variability, 40 randomly selected CT images were re-examined by the same examiner. The intraclass correlation coefficient for ACI was 0.949 [95% confidence interval (CI) = 0.88–0.98]. Median ACI-2016 and ACI-2017 values were evaluated. ΔACI of ≥ third tertile value of this study was defined as high.

### Comparison of clinical characteristics between PPI and non-PPI groups

PPI and non-PPI groups were compared according to age, sex, history of CVD, medication, dialysis vintage, serum data, ACI, and ΔACI. Finally, multivariate logistic regression analysis was performed to determine factors independently associated with higher ΔACI progression (ΔACI ≥ third tertile value of this study).

### Statistical analysis

Statistical analyses were performed using SPSS version 22.0 (IBM Corp., Armonk, NY, USA). Categorical variables are presented as percentages, normally distributed continuous variables as means [standard deviations (SDs)], and non-normally distributed variables as medians [interquartile ranges (IQRs)]. The Wilcoxon signed-rank test was used for comparing ACI-2016 and ACI-2017 values. Sex (0 = female, 1 = male), DMN (0 = other, 1 = presence), current smoking (0 = absence, 1 = presence), history of CVD (0 = absence, 1 = presence), and medication (0 = absence, 1 = presence) were included as binary variables in the model. Comparisons between the PPI and non-PPI groups were performed using the Fisher’s exact test or Chi-square test, Student’s *t*-test (normally distributed data), and Mann–Whitney *U* test (non-normally distributed data). We adopted propensity scores to adjust patient backgrounds due to small sample size. A direct adjustment using propensity score did not compromised statistical power by reducing covariates into a single variable, as reported previously [12, 13]. To adjust the effect of PPIs for vascular calcification, the propensity score was created by a logistic regression providing the predicted probability of PPIs exposure. A dependent variable of PPIs exposure was adjusted by the propensity score including patients’ clinical characteristics (age, sex, DMN, current smoking, history of CVD, antiplatelet agents, and systolic blood pressure) and baseline values of laboratory data (serum albumin, CRP, and high-density lipoprotein cholesterol levels). The propensity score was used as a covariate in the multivariate models evaluating the adjusted effect of each factor. The backward elimination logistic regression analysis was performed to determine the independent predictors for higher ΔACI progression (ΔACI ≥ third tertile value). Based on previous studies, well-known vascular calcification progression factors, such as HD vintage, warfarin, baseline ACI (ACI-2016), annual mean values of Ca × P, i-PTH, and Kt/V were also included in multivariate models. Because serum Mg level is an intermediate variable in the possible causal pathway between PPI use and vascular calcification progression, accounting for the annual mean value of Mg levels after PPIs exposure could mask the true effect of PPI. Therefore, we included baseline Mg level in multivariate models. In addition, we also included H2 antagonist in models to control the confounding factor of usage of acid reducers. According to the guideline of the Japan Society of Dialysis Therapy (JSDT) [14], levels of i-PTH were categorized into 3 groups; low (<60 pg/mL), normal (60–240 pg/mL), and high (>240 pg/mL). These were included as 3 variables in the models as follows; i-PTH (1 = low, 2 = normal, 3 = high). We transformed continuous variables such as HD vintage, Kt/V, baseline ACI (ACI-2016), and baseline serum Mg into binary variables, according to those of median or mean values in all study patients. A higher Ca × P was defined as Ca × P of >55, based on the guideline of JSDT [14]. Odds ratio (OR) with 95% confidence intervals (CIs) associated with each factor was calculated after adjusting for potentially confounding factors. *P* value of < 0.05 was considered statistically significant.

## Results

### Patient classification

All the patients’ clinical characteristics have been summarized in Table 1. We excluded 9 patients who received PPIs for < 12 months during the study period (the mean administration period was 6.6 months). Therefore, this retrospective study included 200 patients on HD (128 males, 72 females) with a median age of 67 (IQR, 58–75) years (Table 1). The mean follow-up period was 12.0 (SD, 1.0) months. The number of PPI and H2-antagonist users was 112 (56%) and 19 (10%), respectively; 69 (35%) did not use acid reducers. Accordingly, the number of patients in the PPI and non-PPI groups was 112 (56%) and 88 (44%), respectively (Fig 1). Median ACI-2016 and ACI-2017 values were 42.0% (17.7–71.4) and 49.6% (23.3–76.7), respectively. ACI-2017 was significantly higher than ACI-2016 (*P* < 0.001). Median and third tertile value of ΔACIs were 4.2% and 5.8%, respectively; a higher ΔACI was defined as ΔACI of ≥ 5.8%.

**Table 1:** Clinical characteristics of the study patients.

	All	PPI	Non-PPI	*P* value
Number	200	112 (56%)	88 (44%)	*-*
Age* (year)	67 (58–75)	69 (59–76)	66 (58–75)	*0*.*250*
Sex (male^‡^), n (%)	128 (64%)	76 (68%)	52 (59%)	*0*.*200*
Cause of CKD				*0*.*140*
DMN^‡^ (presence), n (%)	105 (53%)	64 (57%)	41 (47%)	
Others^‡^ (presence), n (%)	95 (47%)	48 (43%)	47 (53%)	
Modality of HD				*0*.*290*
HD, n (%)	43 (22%)	21 (19%)	22 (25%)	*-*
Hemodiafiltration, n (%)	157 (78%)	91 (81%)	66 (75%)	*-*
Dialysis time* (hours)	4.0 (3.5–4.0)	4.0 (3.5–4.0)	4.0 (3.5–4.0)	*0*.*735*
Baseline Kt/V^†^	1.44 (0.27)	1.44 (0.25)	1.45 (0.30)	*0*.*920*
Annual mean Kt/V^†^	1.44 (0.28)	1.44 (0.26)	1.45 (0.31)	*0*.*850*
Baseline Hemoglobin^†^ (g/dL)	10.9 (1.3)	10.9 (1.4)	10.9 (1.2)	*0*.*820*
Annual mean Hemoglobin* (g/dL)	11.1 (10.4–11.5)	11.1 (10.3–11.6)	11.1 (10.5–11.5)	*0*.*980*
Systolic blood pressure^†^ (mmHg)	153 (21)	154 (21)	150 (22)	*0*.*190*
Diastolic blood pressure^†^ (mmHg)	79 (14)	80 (13)	78 (14)	*0*.*320*
Current smoking^‡^ (presence)	34 (17%)	22 (20%)	12 (14%)	*0*.*260*
History of CVD^‡^ (presence), n (%)	77 (39%)	51 (46%)	26 (30%)	*0*.*021*
HD* vintage (months)	59 (29–111)	55 (25–106)	61 (34–118)	*0*.*190*
PPI (presence), n (%)	112 (56%)	112 (100%)	0 (0%)	*<0*.*001*
H2-antagonist^‡^(presence), n (%)	19 (10%)	0 (0%)	19 (22%)	*<0*.*001*
Antiplatelet agents^‡^(presence), n (%)	94 (47%)	64 (58%)	30 (34%)	*0*.*001*
Furosemide^‡^(presence), n (%)	29 (15%)	14 (13%)	15 (17%)	*0*.*370*
Calcium carbonate^‡^(presence), n (%)	63 (32%)	27 (24%)	36 (41%)	*0*.*011*
Lanthanum carbonate^‡^(presence), n (%)	103 (52%)	59 (53%)	44 (50%)	*0*.*710*
Polymeric phosphate binders^‡^(presence), n (%)	55 (27%)	26 (23%)	29 (33%)	*0*.*130*
Iron-containing phosphate binders^‡^(presence), n (%)	60 (30%)	33 (29%)	27 (31%)	*0*.*850*
Cinacalcet^‡^(presence), n (%)	61 (30%)	32 (29%)	29 (33%)	*0*.*500*
Warfarin^§^ (presence), n (%)	10 (5.0%)	5 (4.4%)	5 (5.6%)	*0*.*700*
Baseline serum albumin* (g/dL)	3.5 (3.3–3.7)	3.5 (3.2–3.7)	3.6 (3.4–3.8)	*0*.*012*
Annual mean serum albumin* (g/dL)	3.5 (3.3–3.7)	3.5 (3.3–3.7)	3.6 (3.4–3.8)	*0*.*005*
BMI* (kg/m^2^)	21.4 (19.4–24.4)	21.5 (19.5–24.7)	21.2 (19.3–23.5)	*0*.*290*
Baseline CRP* (mg/dL)	0.13 (0.04–0.55)	0.21 (0.04–0.56)	0.09 (0.03–0.39)	*0*.*120*
Annual mean CRP* (mg/dL)	0.29 (0.14–0.74)	0.37 (0.16–0.98)	0.24 (0.12–0.59)	*0*.*014*
Baseline high-density lipoprotein cholesterol* (mg/dL)	49 (38–60)	46 (37–59)	53 (41–60)	*0*.*068*
Annual mean high-density lipoprotein cholesterol* (mg/dL)	49 (40–60)	47 (39–57)	54 (44–61)	*0*.*012*
Baseline low-density lipoprotein cholesterol* (mg/dL)	80 (65–97)	78 (65–103)	82 (65–96)	*0*.*590*
Annual mean low-density lipoprotein cholesterol* (mg/dL)	82 (68–98)	82 (68–98)	82 (65–98)	*0*.*650*
Baseline serum phosphate^†^ (mg/dL)	5.4 (1.6)	5.5 (1.7)	5.3 (1.5)	*0*.*400*
Annual mean serum phosphate^†^ (mg/dL)	5.5 (1.1)	5.5 (1.1)	5.5 (1.2)	*0*.*820*
Baseline corrected Ca^†^ (mg/dL)	9.1 (0.6)	9.2 (0.6)	9.1 (0.7)	*0*.*140*
Annual mean corrected Ca^†^ (mg/dL)	9.2 (0.5)	9.2 (0.5)	9.2 (0.5)	*0*.*600*
Baseline Ca × P^†^	49 (15)	50 (15)	48 (14)	*0*.*140*
Annual mean Ca × P^†^	50 (11)	50 (11)	51 (11)	*0*.*990*
Baseline i-PTH* (pg/mL)	138 (80–212)	133 (78–212)	139 (84–213)	*0*.*590*
Annual mean i-PTH* (pg/mL)	152 (107–197)	158 (106–199)	142 (108–194)	*0*.*280*
Baseline serum Mg* (mg/dL)	2.2 (2.0–2.5)	2.1 (1.9–2.4)	2.3 (2.1–2.5)	*<0*.*001*
Annual mean serum Mg* (mg/dL)	2.2 (2.0–2.5)	2.1 (1.9–2.4)	2.3 (2.1–2.5)	*<0*.*001*
ACI-2016* (%)	42.0 (17.7–71.4)	41.7 (21.9–66.5)	44.6 (12.3–73.3)	*0*.*750*
ACI-2017* (%)	49.6 (23.3–76.7)	49.2 (26.7–76.5)	50.8 (15.8–78.3)	*0*.*500*
ΔACI* (%)	4.2 (1.7–7.5)	5.0 (2.5–9.8)	3.8 (0.8–6.5)	*0*.*009*

*Mann–Whitney *U* test

^**‡**^ Chi-square test

^**†**^ Student’s *t*-test

^§^Fisher’s exact test

CKD, chronic kidney disease; DMN, diabetic nephropathy; HD, hemodialysis; CVD, cardiovascular disease; PPI, proton pump inhibitor; H2, histamine-2 receptor, BMI, body mass index; CRP, C-reactive protein; i-PTH, intact parathyroid hormone. Mg, magnesium; Ca, calcium; ACI, abdominal aortic calcification index

### Comparison of clinical characteristics between the PPI and non-PPI groups

**Fig 2:**
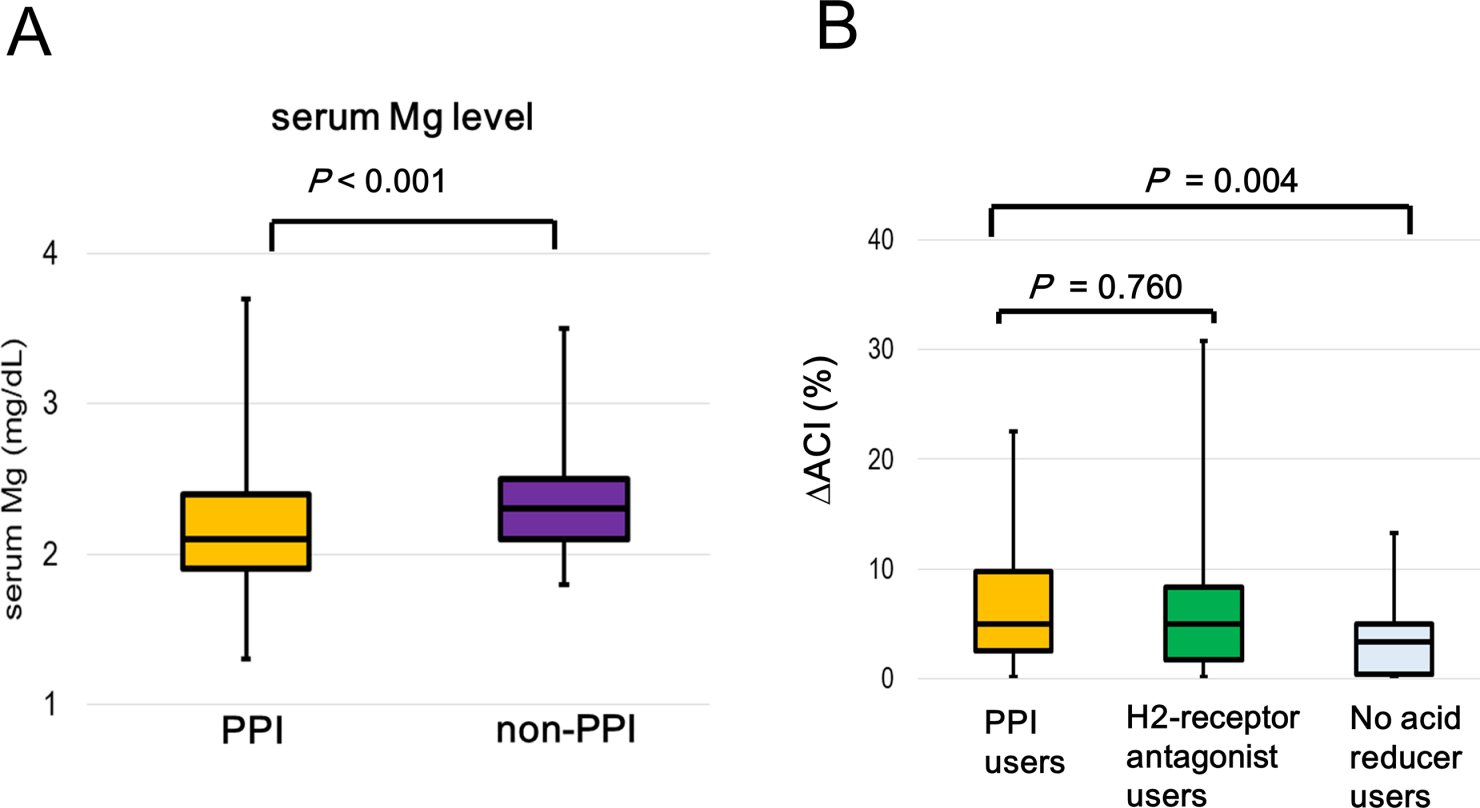
Comparison of clinical characteristics between the proton pump inhibitor (PPI) and non-PPI groups. The proton pump inhibitor (PPI) group had significantly lower levels of serum (magnesium) Mg (A) than those of the non-PPI group. Median values of ΔACIs in the patients on PPIs, histamine-2 receptor (H2) antagonist, and non-acid reducer were 5.0% (2.5–9.8), 5.0% (1.7–8.3), and 3.3% (0.4–5.0), respectively. There was no significant deference in ΔACI between patients on PPIs and those on H2-receptor antagonist (B).

Clinical characteristics, medications, annual mean and baseline values of laboratory data of patients in the PPI and non-PPI groups are presented in Table 1. The number of patients with history of CVD (*P* = 0.021) and using antiplatelet agents (*P* = 0.001) were significantly higher in the PPI than in the non-PPI group. In baseline values of laboratory data, the patients with PPI had significantly lower levels of serum albumin (*P* = 0.012) and serum Mg (*P* < 0.001) than those without PPI. Similarly, the PPI group had significantly lower levels of serum albumin (*P* = 0.005), high-density lipoprotein cholesterol (*P* = 0.012), and serum Mg (*P* < 0.001, Fig 2A) than those of the non-PPI group in annual mean values of laboratory data. The PPI group had significantly higher levels of CRP (*P* = 0.014) and progression of ACI than that in the non-PPI group in annual mean values of laboratory data. No significant deference was observed in ΔACI between PPI users and H2-receptor antagonist users (5.0% vs 5.0%, *P* = 0.760) (Fig 2B).

### Independent risk factors for high ΔACI using the backward elimination logistic regression analysis

**Fig 3:**
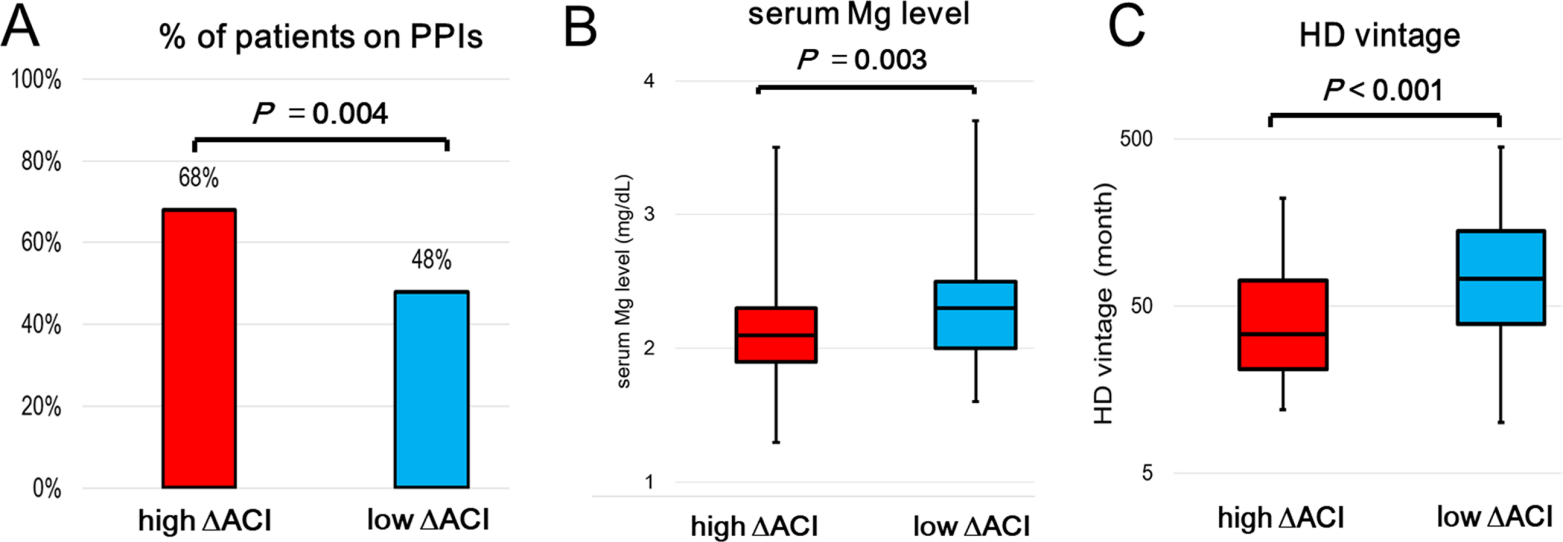
Comparison of clinical characteristics between the high and low ΔACI groups. The high ΔACI group (≥ 5.8%) had a significantly higher number of PPI (A) than in the low ΔACI group (< 5.8%). Annual mean serum magnesium (Mg) level (B) was significantly lower in the high ΔACI than in the low ΔACI group. Hemodialysis (HD) vintage (C) was significantly shorter in the high ΔACI than in the low ΔACI group.

**Fig 4:**
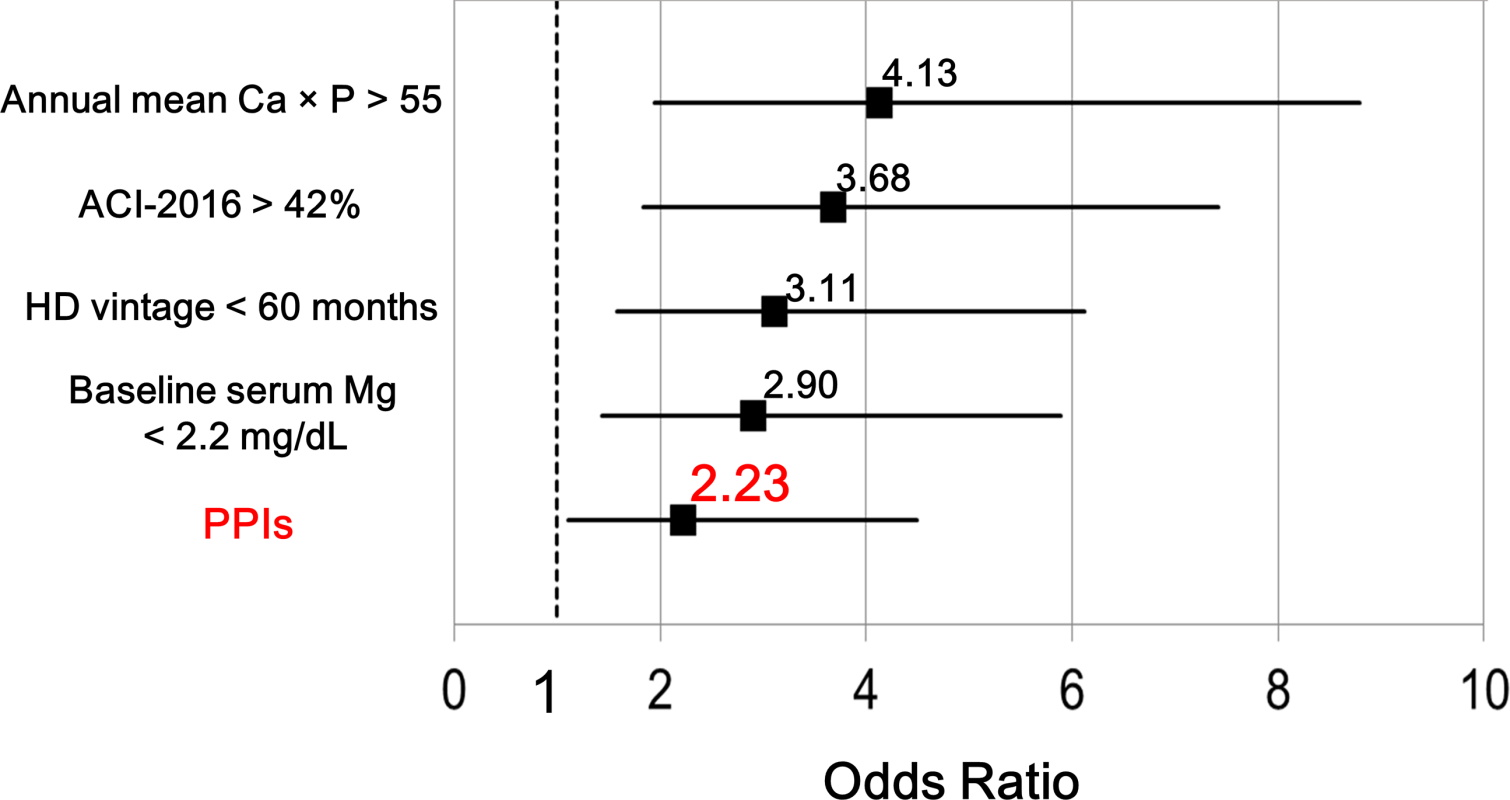
Independent risk factors for high ΔACI using the backward elimination logistic regression analysis. Annual mean calcium phosphorus product (Ca × P) >55, abdominal aortic calcification index in 2016 (ACI-2016) >42%, hemodialysis (HD) vintage <60 months, baseline serum magnesium (Mg) <2.2 mg/dL, and Proton pump inhibitors (PPIs) were independent factors associated with high ΔACI. The propensity score was adopted to include patient backgrounds (age, sex, DMN, current smoking, history of CVD, antiplatelet agents, and systolic blood pressure) and baseline values of laboratory data (serum albumin, CRP, and high-density lipoprotein cholesterol levels) as a single variable for multivariate analysis. We also included annual mean Kt/V >1.44, Histamine -2 (H2)-receptor antagonist, warfarin, annual mean intact PTH (i-PTH), and the propensity score into regression models.

Clinical characteristics, medications, and laboratory data of patients in the high and low ΔACI groups are presented in Table 2 and Fig 3. Independent risk factors for ACI progression rate of ≥ 5.8% were evaluated using the backward elimination multivariate logistic regression analysis (Fig 4), which included annual mean Ca × P >55 (OR = 4.13; 95% CI = 1.94–8.79; *P* <0.001), ACI-2016 >42% (OR = 3.68; 95% CI = 1.83–7.42; *P* <0.001), HD vintage <60 months (OR = 3.11; 95% CI = 1.58–6.12; *P* = 0.001), baseline serum Mg <2.2 mg/dL (OR = 2.90; 95% CI = 1.43–5.89; *P* = 0.003), and PPIs (OR = 2.23; 95% CI = 1.11–4.49; *P* = 0.025) after accounting for confounding factors including the propensity score.

**Table 2:** Clinical characteristics of the high and low ΔACI groups.

	High ΔACI	Low ΔACI	*P* value
Number	77 (39%)	123 (61%)	-
Age* (year)	69 (60–76)	66 (58–74)	*0*.*170*
Sex (male^**‡**^), n (%)	54 (70%)	74 (60%)	*0*.*150*
Cause of CKD			
DMN^**‡**^ (presence), n (%)	47 (61%)	58 (47%)	*0*.*056*
Modality of HD^**‡**^			*0*.*390*
HD, n (%)	19 (25%)	24 (20%)	*-*
Hemodiafiltration, n (%)	58 (75%)	99 (80%)	*-*
Dialysis time* (hours)	4.0 (3.5–4.0)	4.0 (4.0–4.0)	*0*.*001*
Annual mean Kt/V^**†**^	1.38 (0.25)	1.48 (0.30)	*0*.*010*
Annual mean hemoglobin (g/dL)	11.0 (10.3–11.5)	11.1 (10.4–11.5)	*0*.*540*
Systolic blood pressure^**†**^ (mmHg)	153 (18)	152 (23)	*0*.*790*
Diastolic blood pressure^**†**^ (mmHg)	78 (12)	79 (15)	*0*.*770*
Current smoking^**‡**^ (presence)	15 (19%)	19 (15%)	*0*.*460*
History of CVD^**‡**^ (presence), n (%)	30 (39%)	47 (38%)	*0*.*920*
HD* vintage (months)	34 (21–71)	73 (39–141)	*<0*.*001*
PPIs^**‡**^ (presence), n (%)	53 (68%)	59 (48%)	*0*.*004*
H2-receptor antagonist^**‡**^ (presence), n (%)	8 (10%)	11 (9%)	*0*.*730*
Antiplatelet agents^**‡**^ (presence), n (%)	40 (52%)	54 (44%)	*0*.*270*
Furosemide^**‡**^ (presence), n (%)	16 (21%)	13 (11%)	*0*.*046*
Calcium carbonate^**‡**^ (presence), n (%)	23 (30%)	40 (33%)	*0*.*700*
Lanthanum carbonate^**‡**^ (presence), n (%)	43 (56%)	61 (50%)	*0*.*330*
Polymeric phosphate binders^**‡**^ (presence), n (%)	19 (25%)	36 (29%)	*0*.*480*
Iron-containing phosphate binders^**‡**^ (presence), n (%)	25 (32%)	35 (28%)	*0*.*550*
Cinacalcet^**‡**^ (presence), n (%)	19 (25%)	42 (34%)	*0*.*160*
Warfarin^§^ (presence), n (%)	7 (9.1%)	3 (2.4%)	*0*.*036*
Annual mean serum albumin* (g/dL)	3.5 (3.3–3.7)	3.6 (3.4–3.8)	*0*.*043*
BMI* (kg/m^2^)	21.8 (19.7–23.9)	21.1 (19.1–24.6)	*0*.*480*
Annual mean CRP* (mg/dL)	0.38 (0.15–0.86)	0.27 (0.13–0.67)	*0*.*200*
Annual mean high-density lipoprotein cholesterol* (mg/dL)	49 (39–60)	49 (40–61)	*0*.*430*
Annual mean low-density lipoprotein cholesterol* (mg/dL)	88 (66–103)	79 (68–92)	*0*.*094*
Annual mean serum phosphate^**†**^ (mg/dL)	5.7 (1.0)	5.3 (1.2)	*0*.*020*
Annual mean corrected Ca^**†**^ (mg/dL)	9.2 (0.5)	9.2 (0.5)	*0*.*830*
Annual mean Ca × P^**†**^	53 (10)	49 (11)	*0*.*019*
Annual mean i-PTH* (pg/mL)	152 (109–200)	153 (104–195)	*0*.*870*
Baseline serum Mg* (mg/dL)	2.1 (1.9–2.3)	2.2 (2.0–2.5)	*0*.*001*
Annual mean serum Mg* (mg/dL)	2.1 (1.9–2.3)	2.3 (2.1–2.5)	*0*.*003*
ACI-2016* (%)	54.2 (30.4–68.8)	35.0 (11.7–72.5)	*0*.*025*
ACI-2017* (%)	63.3 (41.7–80.4)	39.2 (13.3–74.2)	*<0*.*001*

*Mann–Whitney *U* test

^**‡**^ Chi-square test

^**†**^ Student’s *t*-test

^§^Fisher’s exact test

PPI, proton pump inhibitor; CKD, chronic kidney disease; DMN, diabetic nephropathy; HD, hemodialysis; CVD, cardiovascular disease; H2, histamine-2 receptor, CRP, C-reactive protein; BMI, body mass index; i-PTH, intact parathyroid hormone. Mg, magnesium; Ca, calcium; ACI, abdominal aortic calcification index

## Discussion

After comparing clinical parameters between the PPI and non-PPI groups, we found significant differences in serum Mg levels and ΔACI. Moreover, the key finding of this study was that PPI use is an independent factor for aortic calcification progression even after adjusting for confounding factors. To the best of our knowledge, this is the first study to demonstrate the independent association between PPI use and aortic calcification progression in patients on maintenance on HD.

Patients on HD have an increased risk of gastrointestinal bleeding due to frequent exposure to anticoagulant and/or antiplatelet agents [15]. Acid-suppressive medications, including PPIs, are prophylactically prescribed to patients on maintenance HD to prevent gastrointestinal bleeding [16]. Our results that the proportion of the patients with history of CVD and receiving antiplatelet agents in the PPI group were significantly higher, may reflect these trends. Similar to the results of the present study, a recent cross-sectional study in Japan revealed that 52.3% and 12.1% of patients on HD were PPI and H2 antagonist users, respectively [3]. Our study showed that patients on PPIs had significantly lower serum Mg levels than those not receiving them (2.1 vs. 2.3 mg/dL) in both baseline and annual mean values. This result was consistent with those of several previous reports, thus implying that PPIs reduce serum Mg levels [3, 4]. PPIs induce hypomagnesemia through decreasing active Mg absorption via transient receptor potential melastatin-6 and -7 (TRPM6/7) in the small intestine [17]. Because Mg has been suggested to protect against phosphate-induced vascular calcification [18, 19], lower Mg levels may promote vascular calcification [4, 20]. Indeed, patients on PPIs presented significantly higher rates of ACI progression than those not on PPIs (5.0 vs. 3.8%, Table 1). However, PPI-induced decrease in serum Mg levels may not completely account for this result. Multivariate logistic analysis revealed both PPI and baseline serum Mg level, as one of the independent factors associated with ACI progression.

Vascular calcification is highly prevalent in patients with CKD and those on HD [21, 22]. Both medial and intimal calcification has been associated with vascular calcification progression in patients on HD. Medial calcification has been associated with nontraditional risk factors, such as Ca and P metabolism and malnutrition, whereas intimal calcification has been strongly associated with traditional risk factors, such as male sex, hypertension, smoking, and diabetes mellitus [23]. Vascular endothelial cell injury has been shown to trigger atherosclerosis. A previous study demonstrated that PPIs could directly damage vascular endothelial cells through symmetric dimethylarginine (ADMA) formation [24]. ADMA, a nitric oxide synthase inhibitor, causes nitric oxide reduction, thereby interfering with endothelium-dependent vasodilatation and causing cardiovascular damage. PPIs have been shown to increase the plasma ADMA levels in vitro and in several clinical studies [25–28]. Moreover, previous reports have shown that plasma ADMA levels were positively correlated with coronary artery calcification scores among patients with CKD [26–29]. A study demonstrated that esomeprazole leads to impaired endothelial lysosomal acidification and enzyme activity, which are associated with protein aggregate accumulation, accelerated telomere erosion, and endothelial senescence [10]. Another recent study revealed that PPIs induce the downregulation of anti-atherogenic chemokines in senescent endothelial cells [29]. These findings suggested that PPIs may potentially aggravate intimal calcification via vascular endothelial cell injury. In addition, one study revealed that lansoprazole increased the nuclear accumulation of runt-related transcription factor 2, which is a key transcription factor associated with medial calcification through osteoblast differentiation of vascular smooth muscle cells [30]. The present study demonstrated that patients with rapid ACI progression had significantly higher PPI usage rates (68% vs. 48%, *P* = 0.004; Fig 3A) and lower annual mean serum Mg levels (2.1 vs. 2.3 mg/dL, *P* = 0.003 Fig 3B) than those in patients with slow ACI progression. Our results suggested that in patients on HD, those receiving PPIs had an approximately 2.2-fold higher risk of rapid aortic calcification progression than those not receiving PPIs. Although PPI-induced reduction in serum Mg levels may be associated with ACI progression to some extent, our results suggested that PPIs affect vascular calcification progression in patients on maintenance HD. Few studies have demonstrated the influence of PPIs on vascular calcification; thus, the mechanism underlying this effect remains unclear. Hence, further studies are needed to elucidate the impact of PPIs on vascular calcification.

Our result demonstrated that patients with rapid progression of ACI had significantly shorter HD vintage than with slow progression (34 vs 73 months, *P* <0.001; Fig 3C). The relationship between HD vintage and vascular calcification progression has not yet been fully understood. Previous studies reported the positive correlation between dialysis vintage and vascular calcification progression [31, 32]. However, another study reported the converse relationship [33]. One previous research revealed that the apoptosis of vascular smooth muscle cell was triggered by the initiation of hemodialysis, which caused rapid and extreme vascular calcification [34]. Based on these findings, we speculated that rapid progression of vascular calcification may be occurred in the early transition period following initiation of HD.

Several limitations of the present study should be considered. First, this study was conducted retrospectively at a single center. In addition, its small sample size and selection bias prevented us from reaching definitive conclusions. Second, we could neither evaluate changes in aortic calcification thickness nor distinguish between medial and intimal calcifications due to use of semiquantitative measurements. Third, the employment of a single examiner to measure all ACIs could be construed as a limitation. Fourth, we could not address the duration and exact reason for using PPIs and H2 antagonists, considering that many patients were already prescribed these medicines by other physicians before HD initiation. Our results showed that patients on PPIs had higher proportions of history of CVD and receiving antiplatelet agents. These implied that the main reason for prescription of acid reducers may be prevention of gastrointestinal bleeding. However, the choice of the kind of acid reducers depended on physicians’ preferences, which were not described in the medical records. Finally, we could not clarify the causal relationship between PPIs and aortic calcification progression. Previous retrospective studies demonstrated that PPI users had greater risk of CKD and myocardial infarction, while H2 antagonist users did not have [35, 36]. However, few prospective studies clarifying whether PPI users are at a greater risk of poor clinical outcomes compared with H2-antagonist users and no acid reducers have been performed. It should be noted that the ACI progression rate in patients with H2-antagonist (5.0%) was not significantly different in those with PPI (5.0%) (*P* = 0.760). Although our study could not evaluate the precise mechanism, further study is necessary to address the impact of acid reducers on ACI progression. Despite these limitations, we demonstrated an independent association between PPI and aortic calcification progression in patients on maintenance HD.

## Conclusions

The present study suggested that PPIs may play a role in the progression of aortic calcification in patients on maintenance HD. Further studies should be needed to clarify the impact of PPIs on vascular calcification progression among these patients.

## Supporting information

S1 Dataset S1 Dataset includes all data of this study patients. (XLSX)
